# Integrated analysis of ATAC-seq and RNA-seq reveals the TCP-ARF molecular module related to pathogenic process of phytoplasma infection in *Paulownia fortunei*

**DOI:** 10.1186/s12864-026-12707-w

**Published:** 2026-03-21

**Authors:** Shunmou Huang, Bingbing Li, Yabing Cao, Guoqiang Fan

**Affiliations:** 1 Institute of Paulownia, Henan Agricultural University, Zhengzhou, Henan 450002 China; 2 College of Forestry, Henan Agricultural University, Zhengzhou, Henan 450002 China

**Keywords:** ATAC-seq, ChIP-seq, Histone modification, EMSA, TCP, ARF, Phytoplasma, Witches' broom, ACRs

## Abstract

**Background:**

Witches’ broom is an important disease of the *Paulownia fortunei*. Understanding the pathogenesis of witches’ broom is a prerequisite for its prevention and control. Phytoplasma is the pathogen of Paulownia witches’ broom.

**Results:**

We investigated the changes in chromatin accessibility before and after phytoplasma infection in *Paulownia fortunei* by analyzing the DNA accessibility (ATAC-seq). In phytoplasma-infected *P. fortunei* (PFI) compared to healthy samples (PF), the closed regions of chromatin(1187 regions) were three times more than the open regions (352 regions). Fifty one percent of the accessible chromatin regions were overlapped with either H3K27ac or H3K9ac peaks. The closed regions were enriched in the conserved motif TGGGC[CT] that is recognized by the TCP transcription factor family. The closed regions in PFI are intersected with ARF family gene locus. The gene *PfARF3* was verified to interact with the PfTCP23 transcription factor. The PfTCP23 was predicted to be interacted with the effector pawb44 in the pathogen of phytoplasma.

**Conclusions:**

The phytoplasma infection in *P. fortunei* is involved in the chromatin changes of the DNA accessibility and histone modification. The binding regions of TCP23 were found to be changed mostly in the accessibility between PFI and PF. The TCP-ARF module was found to be the possible regulatory module inducing the crinkled leaf trait.

**Supplementary Information:**

The online version contains supplementary material available at 10.1186/s12864-026-12707-w.

## Background

Phytoplasmas are plant pathogens that induce diseases such as witches’ broom, phyllody, virescence, and changes in leaf shape and color [1]. They infect hundreds of plant species, including fruit, vegetables, and weed plants [2–4]. The phytoplasmas effectors SAP05, SAP11 and SAP54 interact with SPL, GATA, TCP, and MADS transcription factors (TFs) and induce their destabilization [5–7]. Many pathogens are known to trigger epigenetic changes [8], and pathogen-induced epigenetic deregulations can affect host cell function by promoting the host defense or by supporting pathogen persistence [9].

Chromatin accessibility is measurable through ATAC-seq, which is the abbreviation of assay for transposase-accessible chromatin by sequencing [10]. ATAC-seq leverages Tn5 transposase engineered to insert sequencing adapters into regions of accessible chromatin, thus allowing for the mapping of active chromatin regions where transcription factors and other regulatory proteins are likely to bind [11]. ATAC-seq is utilized to discover key transcription factor under biotic stress [12, 13].

In previous studies, the epigenetic changes have been found in DNA methylation, histone modification and chromatin structure in PFI compared to PF [14–16], but the chromatin accessibility study performed between healthy and phytoplasmas-infected plants has not been reported so far. In the study, we investigate chromatin accessibility between healthy and infected hosts with the leaves of *P. fortunei.* The aim was to detect: (1) changes in chromatin accessibility before and after phytoplasma infection; (2) correlations between histone modification and chromatin accessibility; (3) conserved motifs in the PFI-closed regions in infected sample; (4) the transcription factors binding to conserved motifs; and (5) genes that interact with differentially accessible regions (DARs)

## Results

### Chromatin accessibility and TF footprinting

**Fig. 1:**
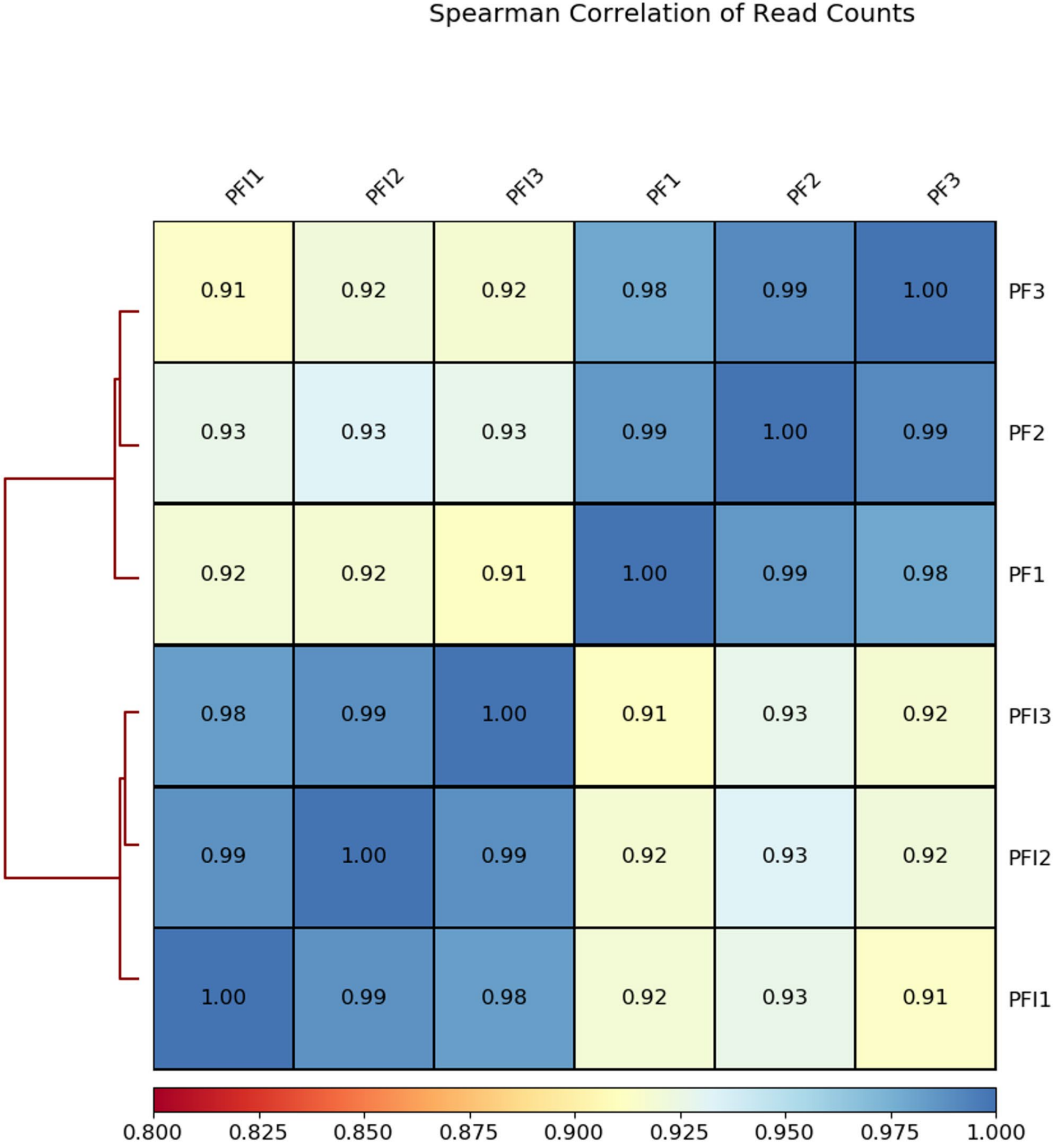
Correlation analysis of ATAC-seq samples. Hierarchically clustered correlation matrix of ATAC-seq replicates (rep1, rep2, and rep3) comparing PF and PFI. Pearson correlations were computed using deepTools by applying multiBamSummary followed by plotCorrelation on read counts divided into 500-bp genomic bins

**Fig. 2:**
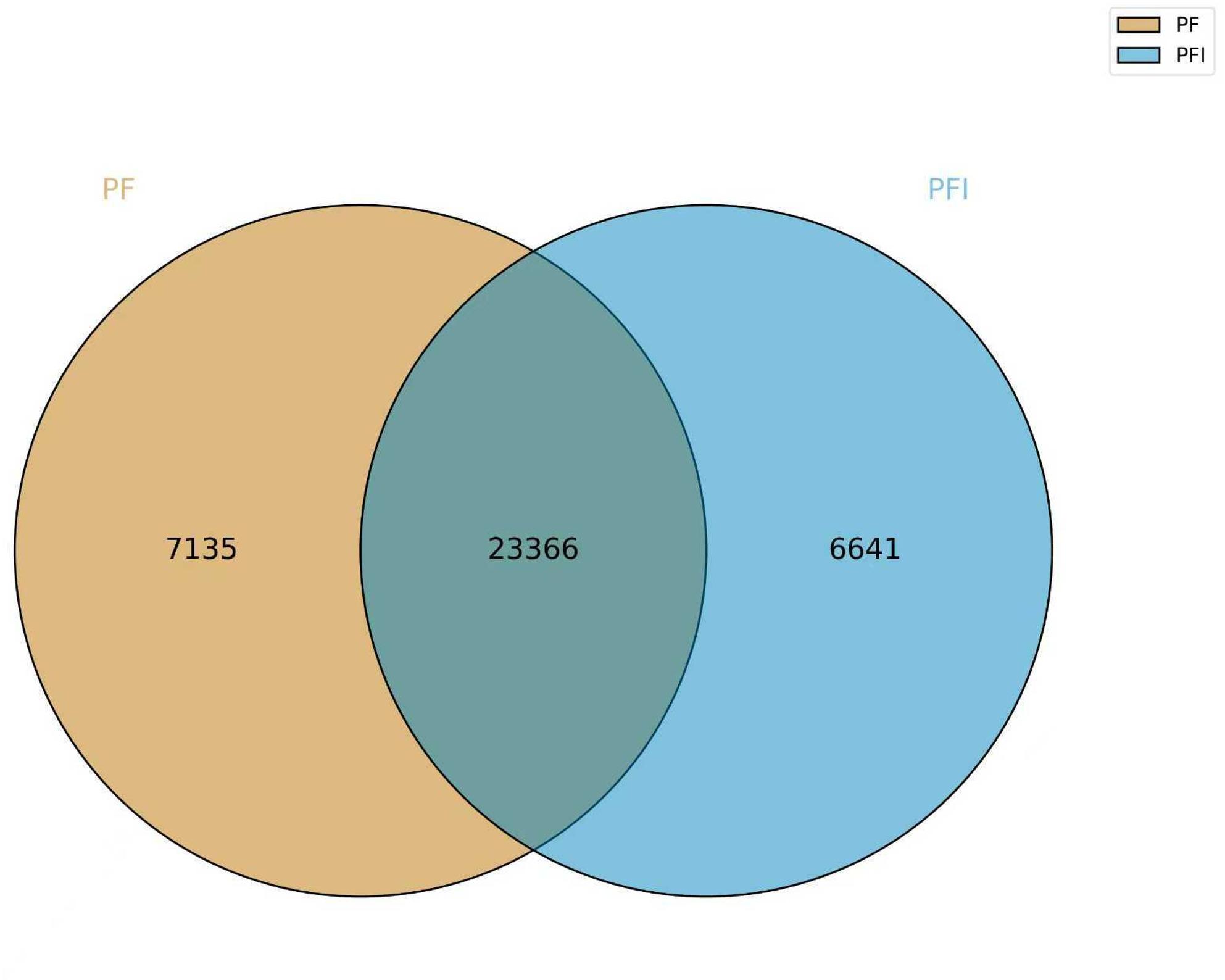
Number of shared and unique peaks between PF and PFI samples. Peaks were identified using uniquely mapped reads with macs2. Peaks overlapping across the genome with peak summits less than 300 bp apart were regarded as identical

**Fig. 3:**
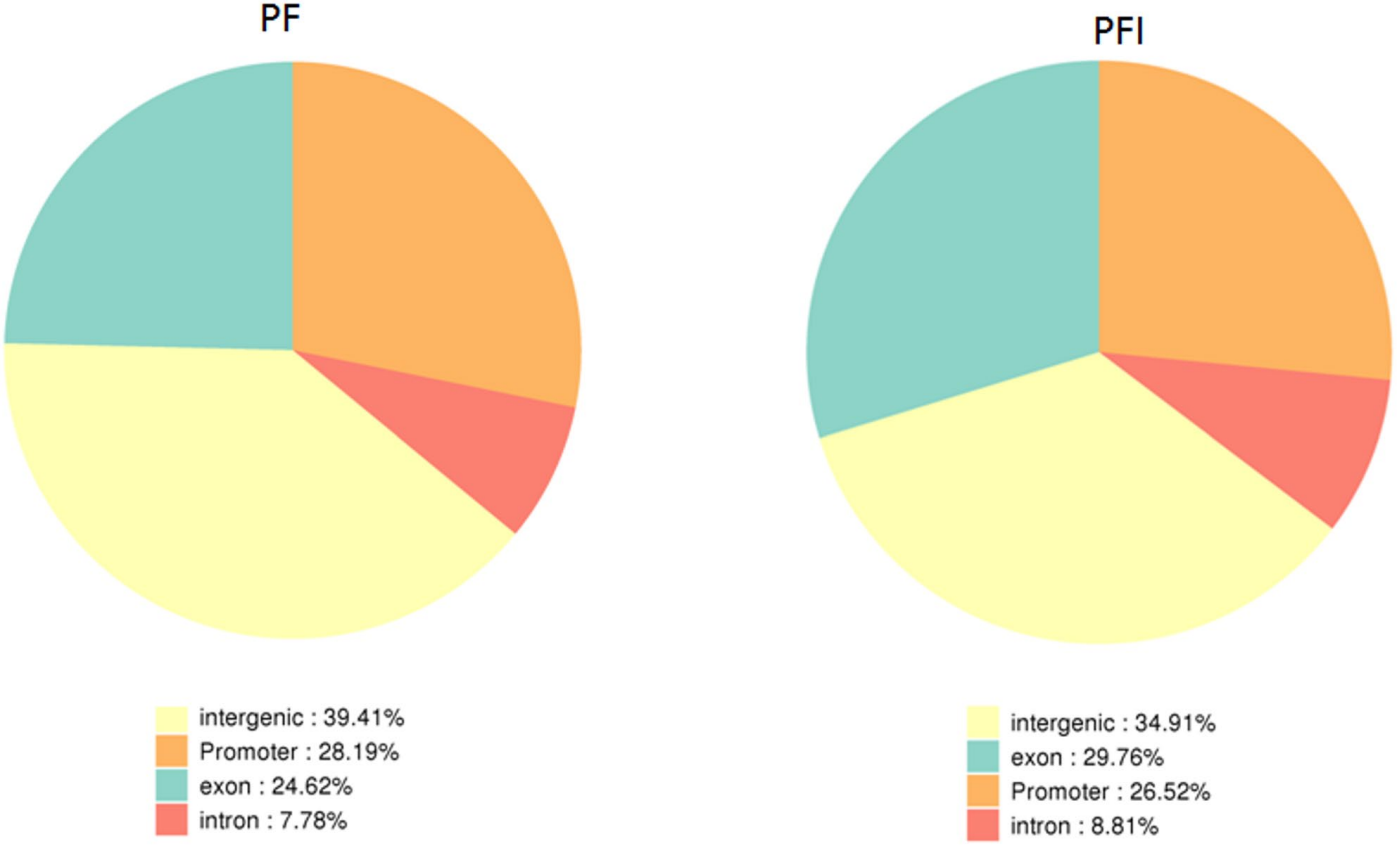
Distribution of chromatin peaks across the genome for PF and PFI samples. The peaks distributed across the annotated genome included promoter, exon, intron and intergenic regions

Chromatin state determines the overall expression of genes in the chromosomes [17]. It dynamically adapts to environmental conditions, encompassing both biotic and abiotic stresses [18–20]. DNA accessibility was hypothesized to be dynamically remodeled during pathogen infection. We utilized ATAC-seq to examine the difference of the chromatin accessibility between healthy and infected *P. fortunei* seeding. The 30-day-old healthy *P. fortunei* (PF) and phytoplasmas-infected *P. fortunei* (PFI) were examined simultaneously, with three biological replicates. High-throughput sequencing of PF and PFI samples on the Illumina X Ten platform yielded 1190 million reads (Table S1). The approximately 200 million reads obtained for every sample were enough for analysis of the open accessibility chromatin of *P. fortunei*. The identified accessible regions showed highly reproducible ATAC-seq peak intensities between biological replicates (R^2^ > 0.98), demonstrating the data’s reliability for evaluating chromatin accessibility (Fig. 1). Statistical analysis showed that the number of accessible chromatin regions was similar between the PF and PFI samples, and most of the peaks were common (Fig. 2). About 70% of ATAC-seq peaks were mainly enriched in intergenic and promoter regions, with a higher concentration in intergenic regions (Fig. 3).

ATAC-seq signals can detect hundreds of TF motifs simultaneously, therefore, we used the FIMO software to perform TF footprint analysis [21]. We downloaded 656 plant TF binding profiles from the JASPAR database [22]. A total of 4,535,171 and 4,274,130 TF footprints were identified from 30,501 to 30,011 accessible chromatin regions (ACRs) in the PF and PFI sample. The most enriched TF binding sites were recognized for the BPC and Dof TF families.

### Differentially accessible regions indicate host genome remodeling under phytoplasmas infection

**Fig. 4:**
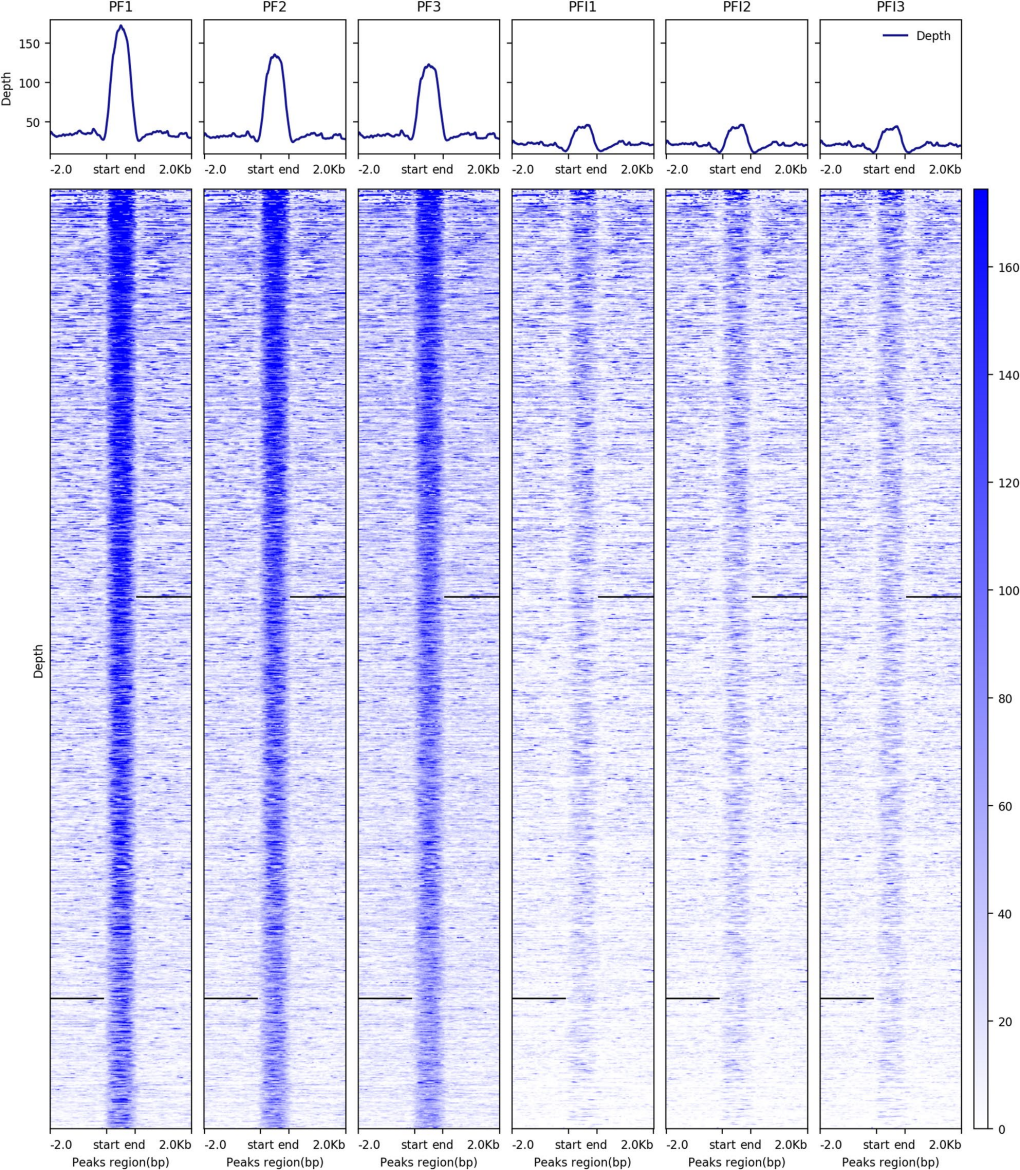
The differentially accessible regions between PF and PFI. The more accessible peaks in PF. Each row represents one peak region. The color represents the intensity of chromatin accessibility

**Fig. 5:**
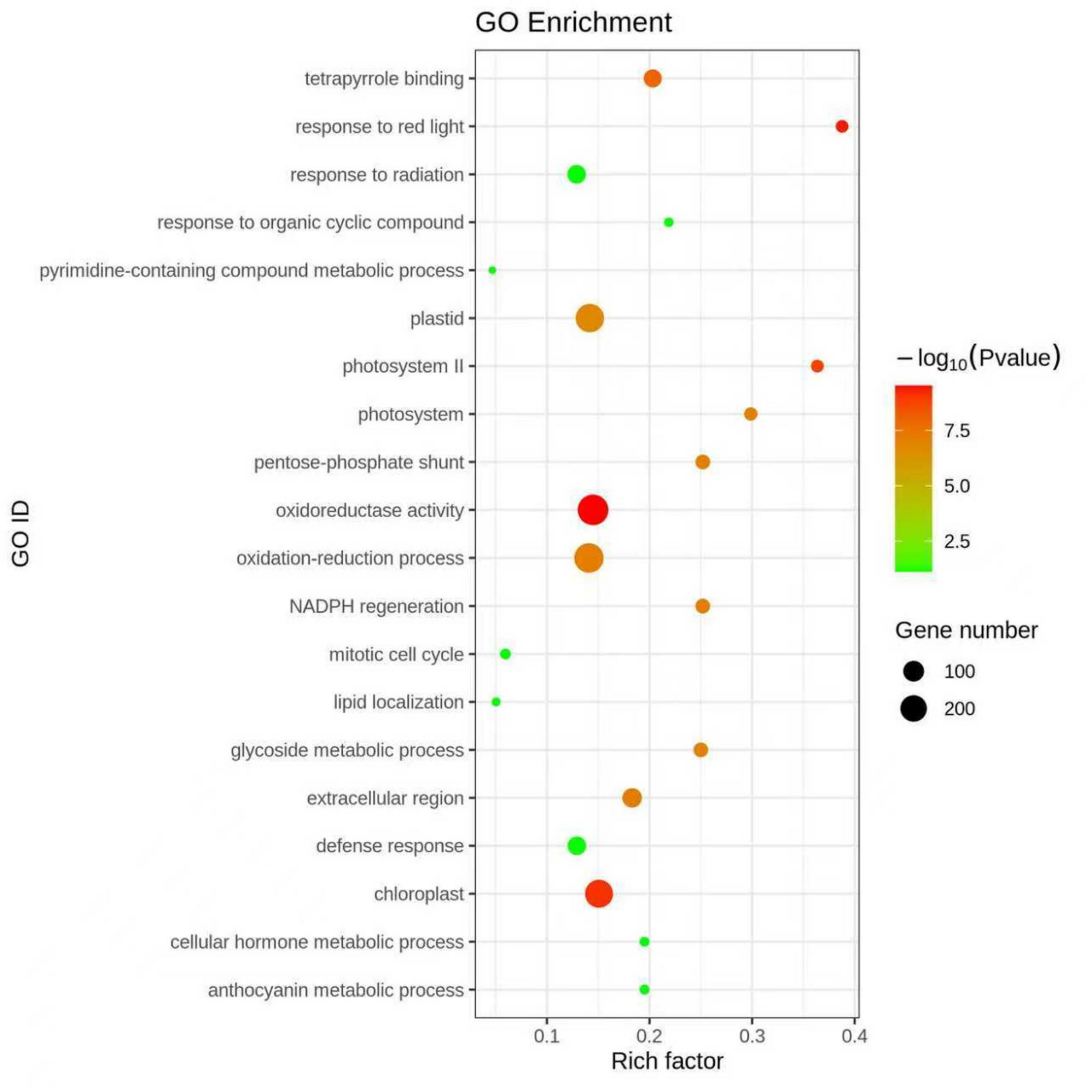
The enriched terms in the genes associated with more open chromatin in the PF than PFI sample

We quantified differences in accessible chromatin regions between PF and PFI samples via calculating total read counts at each ATAC-seq peak in the combined peak sets. We then evaluated the read counts using edgeR to identify quantitative differences in accessible regions between the two samples [23]. Peaks with log fold change > 2.0 and with significant q-value (< 0.05) were categorized as DARs. We named the more accessible regions (MARs) in PF or PFI as PF-MARs or PFI-MARs, respectively. We identified a total of 1187 PF-MARs and 352 PFI-MARs respectively (Fig. 4, Table S2). To reveal the roles of PF-MARs in phytoplasma infected response, DEGs within 100 kb upstream and downstream of PF-MARs were annotated [24]. The DEGs were from the RNA-seq datasets reported previously [25]. We found that 3157 (44%) of the 7090 DEGs were intersected with the PF-MARs, whereas only 739 (10%) DEGs were in the PFI-MARs. These DEGs intersected with PF-MARs were enriched in oxidoreductase activity and defense response (Fig. 5, Table S3).

### Chromatin accessible regions coexisted with histone modification

**Fig. 6:**
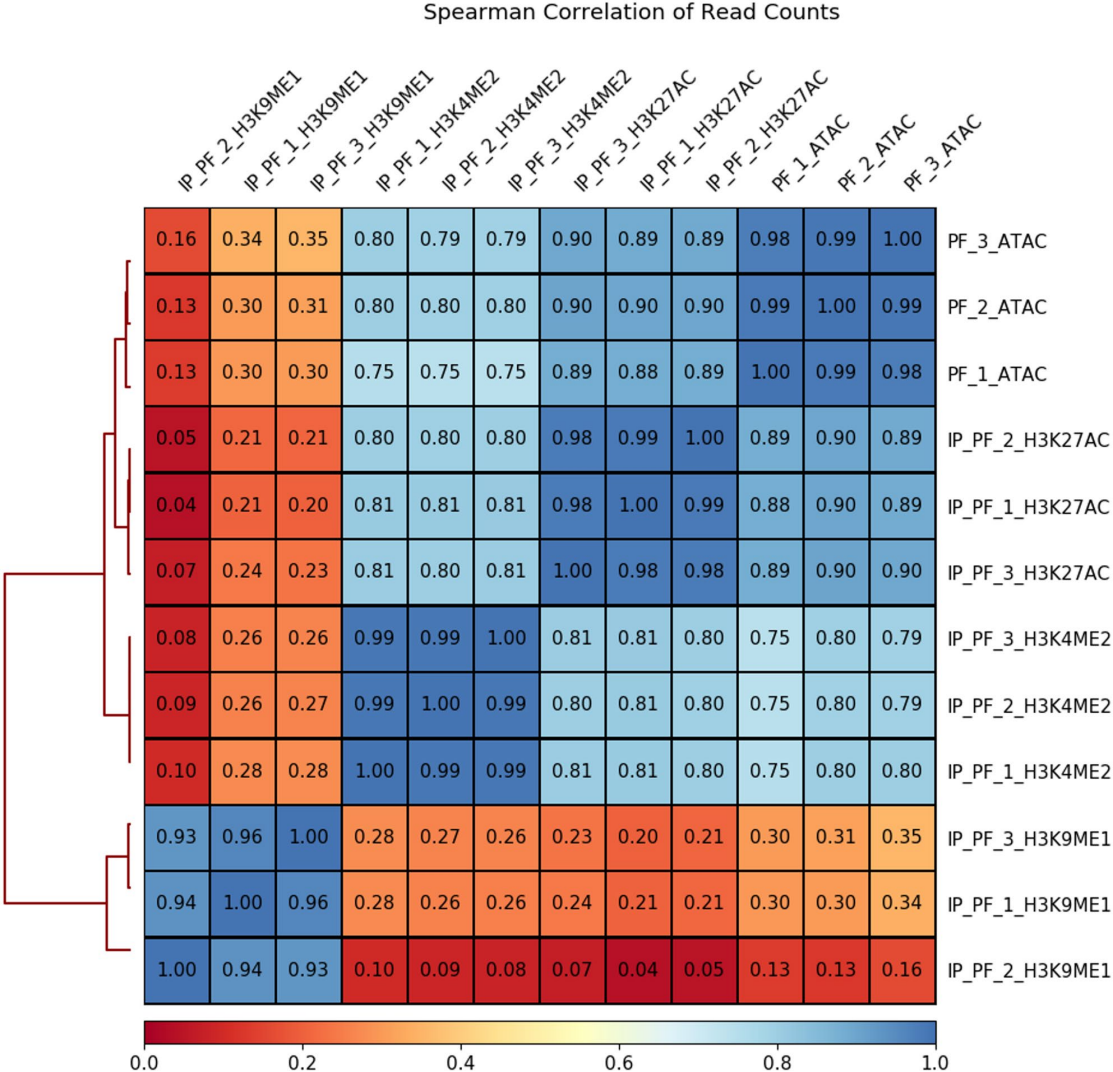
Analysis of the correlation between ATAC-seq and histone ChIP-seq data. A hierarchically clustered correlation matrix was constructed for ATAC-seq replicates (rep1, rep2, and rep3) alongside ChIP-seq data for H3K27ac, H3K4me2, and H3K9me1 histone modifications. Pearson correlations were computed using deepTools by applying multiBamSummary followed by plotCorrelation on read counts divided into 500-bp genomic bins

**Fig. 7:**
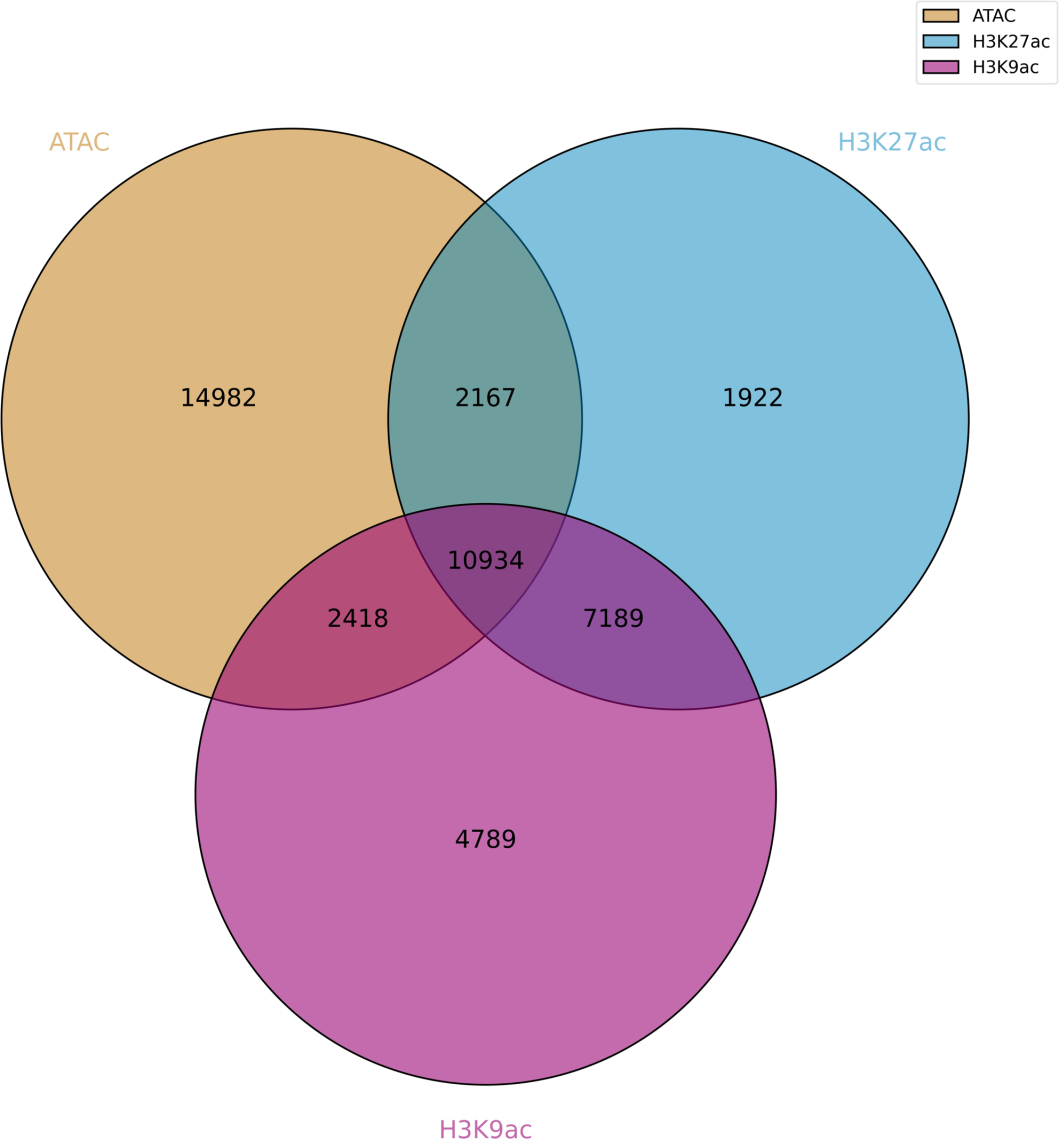
Accessibility chromatin regions overlapped with H3K27ac and H3K9ac histone modification

Research indicates significant alterations in histone H3 epigenetic modifications in *P. fortunei* infected by phytoplasmas [15]. We reanalyzed the H3K9ac, H3K27ac, H3K4me2, and H3K9me1 ChIP-seq datasets for the two samples, with three replicates. The clean reads, obtained after removing contaminated sequences and low-quality reads, were aligned to the *P. fortunei *reference genome. The unduplicated mapped reads were used to call peaks, and correlations among the different marks were determined (Fig. 6). A correlation matrix of chromatin accessibility signals and histone modification signals was generated to detect mutual relationships. We found that the H3K27ac and H3K4me2 marks had more overlaps than the H3K9me1 marks with accessible regions. The IDR software was used to merge the peaks from the three replicates. In the PF dataset, 27,001 H3K9ac, 20,579 H3K27ac, 29,079 H3K4me2, and 7,221 H3K9me1 marks were identified. Of the ATAC-seq peaks, 43.8% overlapped with H3K9ac, 43.0% overlapped with H3K27ac, 30.2% with H3K4me2, and 2.9% with H3K9me1 marks. Fifty one percent of the ATAC peaks were present in either H3K27ac or H3K9ac marks (Fig. 7).

### Comprehensive motif analysis and identification of related transcription factors

**Fig. 8:**
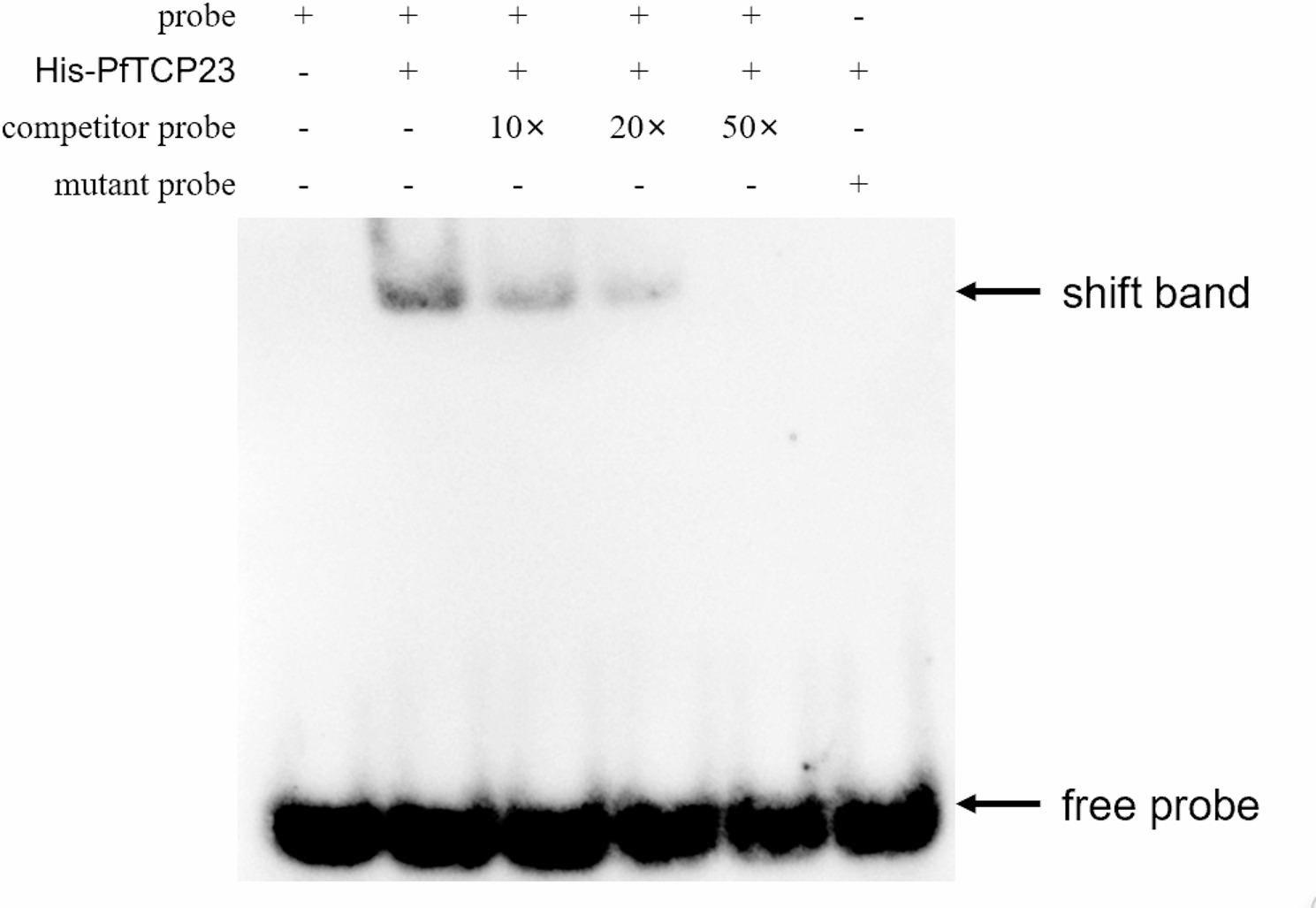
Analysis of PfTCP23 target genes by EMSA. EMSA results demonstrate the in vitro binding of PfTCP23 to GGNCCCAC probes. The arrow marks the location of the protein-DNA complex following the incubation of the biotin-labeled DNA probe with the PfTCP23 protein

Regions with high accessibility are likely to recruit TFs that can regulate the expression of nearby genes [26]. We analyzed sequence motifs overrepresented in PF-MARs and PFI-MARs to pinpoint transcription factors potentially crucial in *P. fortunei’s* response to phytoplasma infection. We discovered 107 and 7 motifs that were overrepresented in PF-MARs and PFI-MARs, respectively (Tables S4-5). The most enriched motif in PF-MARs was (TGGGC[C/T]). The TomTom software identified the motif of the AtTCP23 gene of Arabidopsis thaliana in the JASPAR database was the most similar to the (TGGGC[C/T]) motif identified in the study [27]. Via the EMSA experiment, it was verified that there was a direct interaction between the motif sequence TGGGC[C/T] and PfTCP23 (Fig. 8).

**Fig. 9:**
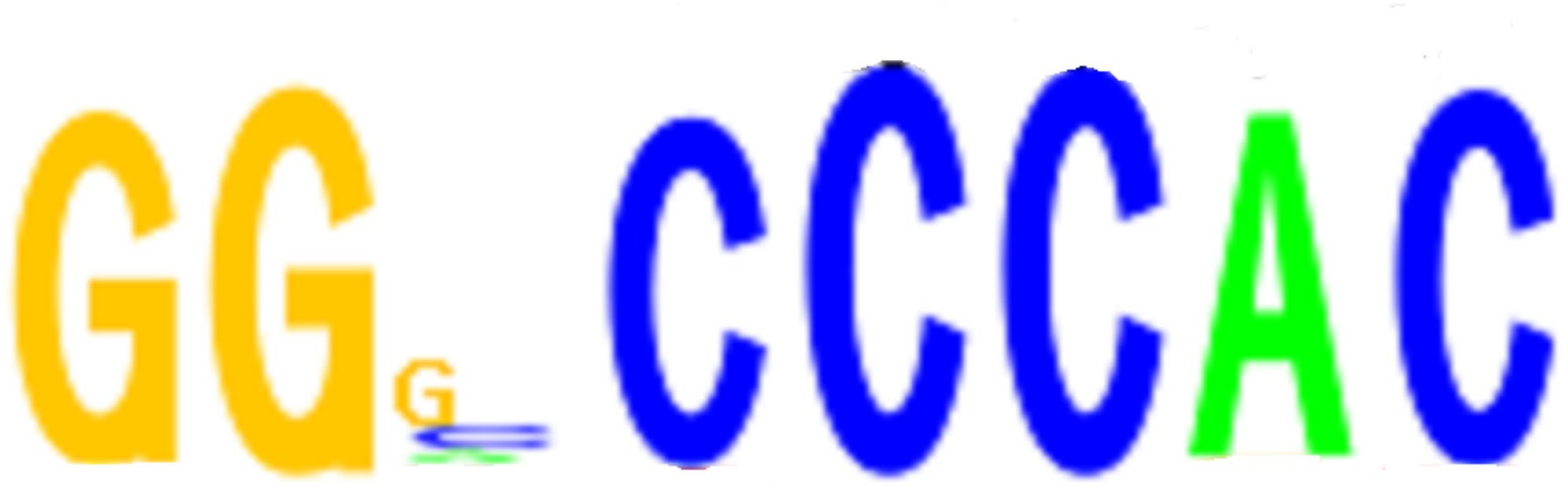
PfTCP23 binding to the GGNCCCAC core motifs. Top top motifs identified via DAP-seq data using the software GEM for analysis

We conducted DNA affinity purification sequencing (DAP-seq) to identify genome-wide binding sites of PfTCP23 and elucidate its function [28]. We identified 8307 enriched peaks with fold enrichment ≥ 10 by analyzing the DAP-seq data, among them, 2130 (25.64%) peaks were located in the promoter regions of annotated genes. The study identified 575 genes through DAP-seq that overlapped with phytoplasma-responsive DEGs, with 348 being upregulated and 227 downregulated. The DAP-seq data showed that the top motif for PfTCP23-binding was GTGGGNCC (Fig. 9). By screening the binding target genes of the PfTCP23 TF, we focused on PfARF3 because of its known interaction with the IAA TF gene family.

### PfTCP23–PfARF3 regulatory module

**Fig. 10:**
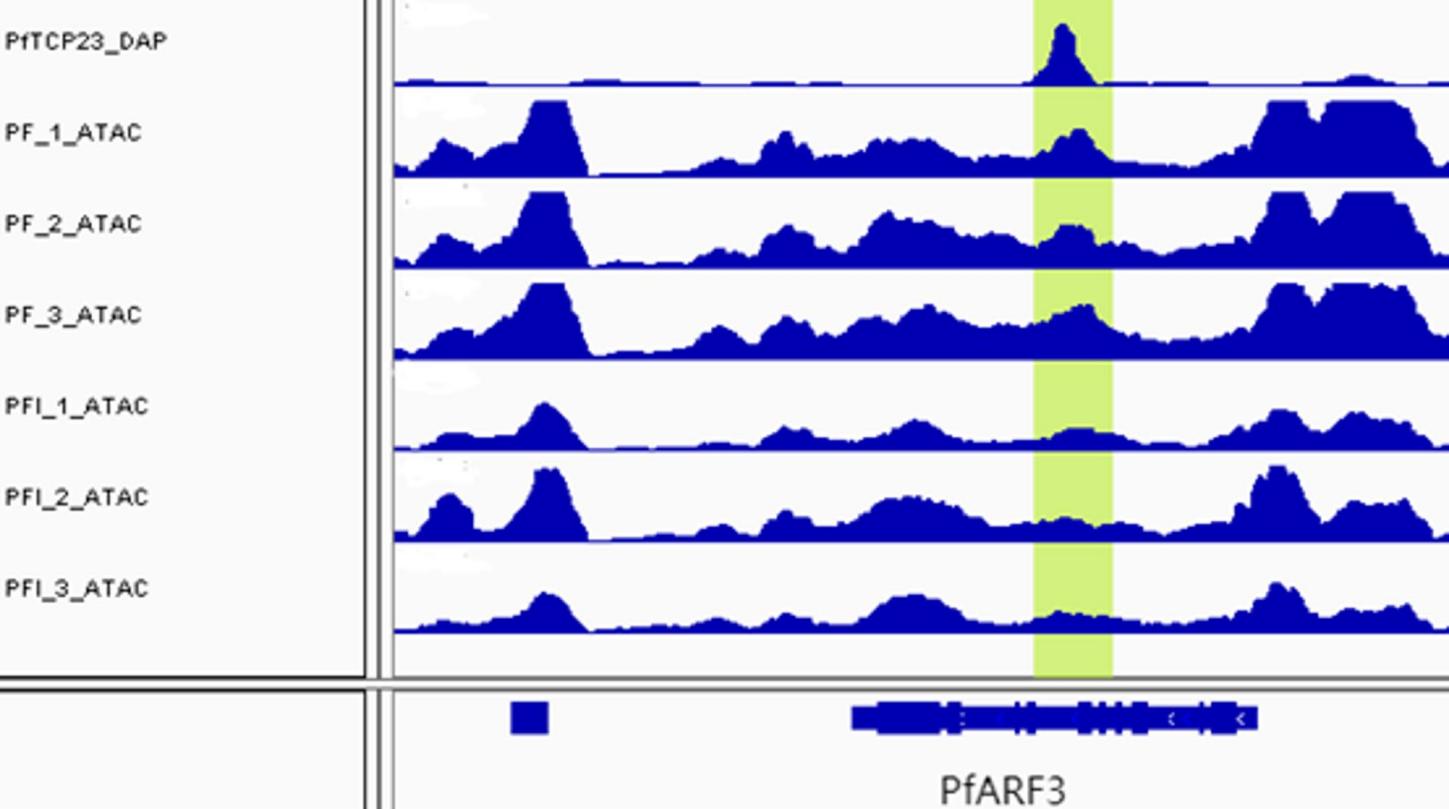
The enriched peaks in the gene PfARF3. IGV screenshot showed one PfTCP23 DAP-seq enriched peak in the target gene of PfARF3. There was also a differentially accessible peaks for ATAC-seq between PF and PFI samples. The green background color indicates the enriched peak

Wrinkled leaf is an important symptom for phytoplasmas diseases [29]. In *A. thaliana*, *ARF3* was identified as the gene for the abaxial domain of the leaf [30]. Abnormal *ARF3* expression induced wrinkled leaf [31]. The homologous gene *PfARF3* in *P. fortunei* was significantly differentially expressed between the PFI and PF samples. One enriched peak was found in the intron region of the gene PfARF3, and this region was in one of the DARs between the PFI and PF samples (Fig. 10). We hypothesized that the wrinkled leaf symptom for phytoplasmas diseases may be caused via the PfTCP23–*PfARF3* regulatory module.

### Interaction prediction via Alphafold3m model

**Fig. 11:**
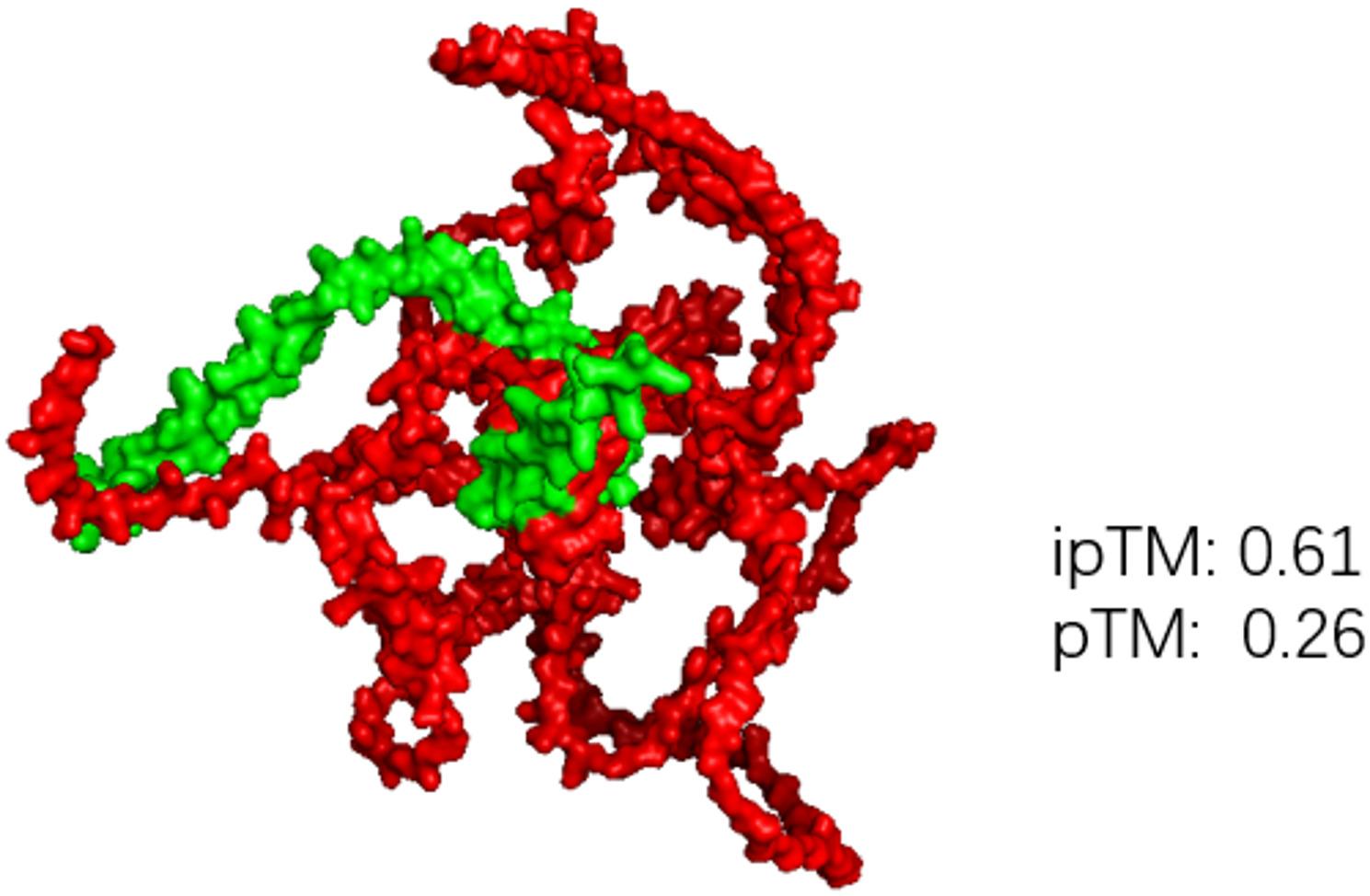
The 3D structure predicted confidently using AlphaFold3. The sum of ipTM and pTM is 0.87 (> 0.75) which means high likelihood of interaction between the effector pawb44 and transcription factor PfTCP23 in host. The green indicates the effector pawb44 and the red indicates the protein PfTCP23

**Fig. 12:**
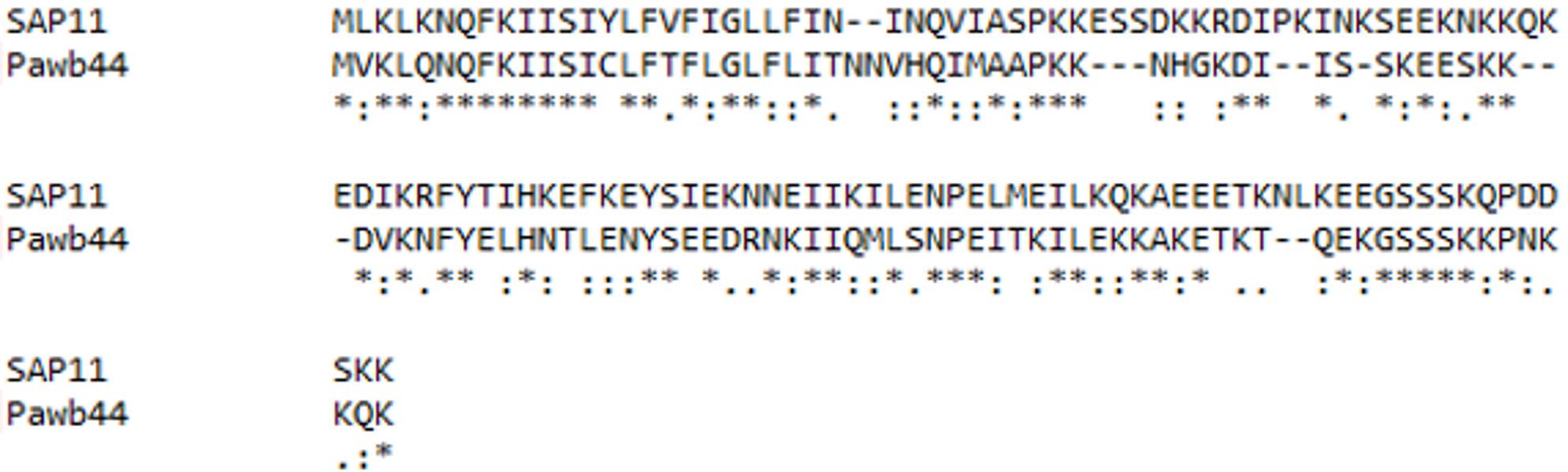
The comparison results between Pawb44 and SAP11

AlphaFold3 was used to model the interaction between the effector pawb44 and PfTCP23. The sum of ipTM and pTM is 0.87 (> 0.75), which means the possibility was very high for the interaction between the effector pawb44 and PfTCP23 (Fig. 11). The pawb44 was the most similar effector in PaWB to SAP11 in AYWB (Fig. 12).

## Discussion

Transcription regulation involves two key processes: the loosening of chromatin structure through chromatin-remodeling complexes and histone post-translational modifications [32–34], and the assembly of transcriptional machinery and RNA polymerase II at the promoter region to initiate transcription [35]. In this study, we investigated chromatin changes in *P. fortunei *related to the pathogenesis of phytoplasmas. We performed ATAC-seq to analyze chromatin changes mediated by phytoplasmas infection. We discovered that many DARs were induced in response to phytoplasmas infection. These findings highlight the impact of phytoplasma infection on chromatin accessibility dynamics and enhance research on phytoplasma-host interactions, particularly the epigenomic mechanisms involved.

We identified 1187 PF-MARs and 353 PFI-MARs, which indicates that DNA regions were less accessible in the PFI sample and that the chromatin tended to close during phytoplasmas infection. Only parts of the PF-MARs overlapped with differentially modified histone regions, implying that these PF-MARs may be induced by the histone modifications [36]. The most enriched motif in the PF-MAR region was TGGGC[C/T]. This motif is recognized by most members of the TCP TF family in *A. thaliana*, with AtTCP8 and AtTCP22 as the exceptions [37]. The TCP gene family, present in various plant species, plays a crucial role in regulating plant architecture and serves as a mechanism for evolutionary influence on plant diversity [38, 39]. The AtTCP18 gene in the TCP family in *A. thalian*a has been related to branching [40], and AtTCP14 and AtTCP15 are related to internode length and leaf shape [41]. The impacted traits are similar to symptoms that are induced by pathogenic phytoplasmas [42]. Phytoplasmas virulence effectors have been shown to interact with TCP family genes that were destabilized after binding the effectors [6, 43, 44]. The effector SAP11 from AYWB have been verified to be interacted with CYC/TB1 TCPs in arabidopsis and destabilized the AtTCP18 gene [45]. Aguilar-Mart í nez et al. [46] showed that AtTCP18 interacts with auxin and SL pathways to modulate branching. The direct target of AtTCP18 were enriched in abscisic acid-related components [47–50]. Idan et al. found that the CIN-TCP gene interacted with the Arabidopsis BRAHMA gene [51]. The remodeling complex including the BRAHMA gene is related to chromatin openness [52]. Under phytoplasmas infection, the destabilized TCP protein may bind to the cis-regulatory elements of downstream genes and down-regulate the target genes, triggering the closed chromatin.

We speculated that the TCP gene *PfTCP23* mediated the pathogenic process under phytoplasmas infection, but which downstream genes were impacted when *PfTCP23* was knocked down is not known. The DAP-seq analysis of downstream genes identified *PfARF3* as one of the target genes of PfTCP23, which is known to be associated with crinkle leaf [31]. In the future, we will block the pathogenic process via bioengineering approach [53].

NLR genes are important components of the process that leads to effector-triggered immunity [54]. We identified seven NLR genes (PfNLR18, PfNLR21, PfNLR23, PfNLR26, PfNLR47, PfNLR160 and PfNLR164) that had lower expression in PFI than they had in PF and were located in relatively low ATAC-seq peaks near the transcription start site in PFI [55]. NLR genes enhance plant immunity by activating robust host defense upon detecting specific pathogen effectors [56]. The results indicate that phytoplasmas infect their hosts by repressing the expression of NLR genes [57]. Dodds et al. discovered that the effector AvrL567 interacted with NLRs to induce a hypersensitive response [58]. Whether NLR genes interact with phytoplasmas effectors requires further validation.

## Conclusions

Our study identified significant DNA accessible changes between PFI and PF samples. A total of 1539 DARs were found between PFI and PF. The most enriched motif is GGNCCCAC recognized by TCP family transcription factors. Fifty one percent of ACRs are regions that overlap with peaks from the H3K27ac or H3K9ac modification. The regulatory module of TCP-ARF was validated to exist.

## Methods

### Plant materials

This study utilized 30-day-old tissue-cultured plantlets of both healthy and phytoplasma-infected P. fortunei (PF and PFI, respectively). The plantlets were cultivated and validated following the method outlined by Fan et al. [59–61]. Terminal buds from both PF and PFI plantlets were collected. For ATAC-seq and ChIP-seq library construction, three independent biological replicates were prepared.

### Transposase-accessible chromatin assay (ATAC) and library preparation

Around 0.5 g of terminal buds were taken from PFI and PF and promptly ground in 2 ml of chilled lysis buffer. The mixture was filtered twice using miracloth and then applied onto 2 ml of dense sucrose buffer in a 10-ml Falcon tube. The nuclei underwent centrifugation at 2200 × g for 15 min at 4 °C, followed by resuspension of the pellets in 500 µl of pre-chilled lysis buffer. The transposition reaction mixture comprised 25 µl of reaction buffer, 2.5 µl of Nextera Tn5 transposase, and 22.5 µl of nuclease-free H2O. The crude nuclei were resuspended in the transposition reaction mixture and incubated at 37 °C for 30 min. A Qiagen MinElute PCR Purification Kit was used to purify the DNA after transposition. The DNA underwent amplification for 10–15 cycles using NEBNext High-Fidelity 2× PCR Master Mix. Amplified libraries were purified with a Qiagen MinElute PCR Purification Kit. The library was eluted using 20 µl of Elution Buffer. Library quality was evaluated using an Agilent 2100 Bioanalyzer and a Qubit fluorometer.

### Chromatin immunoprecipitation-based library preparation

Three grams of *P. fortunei* leaves were washed twice with cold phosphate-buffered saline, cross-linked with 1% formaldehyde for 10 min at room temperature, and then quenched with glycine. Samples were lysed and the chromatin was obtained on ice. The chromatin was sonicated to obtain soluble, sheared chromatin. Subsequently, 20 µl of chromatin was reserved at -20 °C for input DNA, while 100 µl was allocated for immunoprecipitation using H3K27ac, H3K4me2, and H3K9me1 antibodies. Antibody immunoprecipitation was conducted overnight at 4 °C using 10 µg of antibody. The following day, 30 µl of protein beads were added to the samples and incubated for 3h. The beads were then washed sequentially, once with a solution of 20 mM Tris/HCl, 50 mM NaCl, 2 mM EDTA, 1% Triton X-100, and 0.1% SDS; twice with 10 mM Tris/HCl, 250 mM LiCl, 1 mM EDTA, 1% NP-40, and 1% deoxycholic acid; and twice with 1× TE buffer (10 mM Tris-Cl, pH 7.5)0.1 mM EDTA). The bound material was eluted from the beads using 300 µl of elution buffer (100 mM NaHCO3, 1% SDS), followed by treatment with RNase A for 6 h at 65 °C and proteinase K overnight at 45 °C. The sequencing libraries were constructed from immunoprecipitated DNA using the I NEXTFLEX ChIP-Seq Library Prep Kit for Illumina^®^ Sequencing and sequenced on an Illumina X Ten platform with the PE 150 method.

### Protein expression and DNA library construction

The coding sequence of PfTCP23 was cloned into a HaloTag T7 SP6 Flexi expression vector. The Halo–PfTCP23 fusion gene was expressed in a 50 µl reaction incubated for 2 h at 37 °C, as per the manufacturer’s instructions. The fusion proteins were captured directly using Magnetic Halo-Tag Beads. Adaptor-ligated gDNA fragments (50 ng) were incubated with protein-bound beads in 50 µl of wash/bind buffer on a rotator for 1h at room temperature. Beads underwent three washes with the wash buffer to eliminate unbound DNA fragments. The Halo-Tag beads were resuspended in 30 µl of elution buffer and heated at 98 °C for 10 min to denature the protein and release the bound DNA fragments. The supernatant was transferred to a new well, and 25 µl was used in a 50-µl PCR using a KAPA HiFi HotStart ReadyMix PCR Kit for 10 cycles.The obtained DNA libraries were sequenced.

### High-throughput sequencing

Sequencing was performed using the Illumina HiSeq X Ten platform. Libraries were pooled and sequenced together in a single flow cell using paired-end 150-nucleotide reads.

### Computational analysis

For all the analysis, the *P. fortunei* genome sequence and annotations from the NCBI were used as the references. The data for chromosomes 1 to 20, excluding unanchored scaffolds, were used for all the analysis.

### ATAC-seq data analysis

Trimmomatic was used to remove adapter sequences and low-quality reads [62]. Bowtie2 was used to map clean reads to the reference genome, selecting the best match [63]. PCR duplicates were eliminated using SAMtools [64], and peaks were identified with MACS2 software employing default settings [65]. The ATAC-seq peaks that differed between the PF and PFI samples were identified using Diffbind [66]. Gene Ontology (GO) and Kyoto Encyclopedia of Genes and Genomes (KEGG) enrichment analyses were conducted using ClusterProfiler [67]. A heatmap of regulatory region distribution was obtained using Deeptools [68]. Enriched motifs were identified using HOMER [69]. TF footprinting analysis utilized TOBIAS [70], incorporating aligned reads, peaks, and JASPAR TF motifs [22]. TOBIAS corrects Tn5 bias, calculates footprint scores for each base within peaks, identifies TF binding sites using specified TF motifs, and classifies these sites as bound or unbound based on the footprint scores.

### Analysis of ChIP-seq datasets

Sequencing data quality filtering followed the methodology outlined in the ‘Analysis of the ATAC-seq data’ section. BWA was used to align the reads to the reference genome [71]. Reads not uniquely mapped were excluded using SAMtools with a MAPQ threshold of 20 [64]. MACS2 was used to call peaks for each biological replicate, applying a q-value threshold of 0.01 [65]. During the peak calling, input data were used as controls for the ChIP-seq. Only peaks with ≥ 70% overlap between replicates were used for the subsequent analysis. For three replicates, overlapping peaks were initially identified in two replicates, followed by comparison of the third replicate to these integrated peaks.

### Analysis of the RNA-seq dataset

Trimmomatic was used to remove adapter sequences and low-quality reads [62]. TopHat2 was used to align clean reads to the reference genome [72]. The Cufflinks pipeline was utilized to calculate transcript assembly and gene expression levels for each replicate [73]. Genome coverage in reads per million was determined using BEDTools [74].

### Analysis of the DAP-seq data

Bowtie2 was used to align reads to the reference genome [63]. MACS2 was utilized for peak calling [65]. PeakAnnotator was used to analyze the association of DAP-seq peaks within 2 kb upstream of the transcription start site, utilizing GFF files [24]. FASTA sequences were extracted with BEDTools for motif analysis [74]. Motif discovery was conducted utilizing both HOMER and GEM [75].

### Electrophoretic mobility shift assay

Electrophoretic mobility shift assay (EMSA) is a technique used to study protein-DNA interactions by observing the mobility changes of DNA-protein complexes during electrophoresis. The open reading frames of PfTCP23 were cloned into the PATX–MBP vector and expressed to obtain MBP-PfTCP23 fusion proteins. Sangon Biotech synthesized biotin-labeled probes for PfARF3. The EMSA was performed with a LightShift Chemiluminescent EMSA kit.

### Structure modeling and interaction prediction

The locally deployed AlphaFold3 was used to model the interaction between effector from the phytoplasma and protein from the host [76]. The default seed was set to 1. For each modeling run, only the top-ranked model (model_0) was retained and processed. The ipTM and pTM values were extracted from the AlphaFold3 JSON files containing model metadata using custom scripts and ploted in R. IpTM and pTM’s sum exceeding 0.75 indicates a very high likelihood of interaction between the two proteins[77]. The structures were visualized using PyMOL software [78].

## Supplementary Information

Supplementary Material 1.

Supplementary Material 2.

Supplementary Material 3.

Supplementary Material 4.

Supplementary Material 5.

Supplementary Material 6.

## Data Availability

The ATAC-seq, ChIP-seq and DAP-seq data generated in this study have been submitted to the NCBI BioProject database (https://www.ncbi.nlm.nih.gov/bioproject/?term=PRJNA1012301) under accession number PRJNA1012301. The RNA-seq data sets were obtained from the NCBI Sequence Read Archive (SRA) database (https://www.ncbi.nlm.nih.gov/bioproject/PRJNA624264) under accession number PRJNA624264 [79], SRR11787883, SRR11787894, SRR11787905, SRR11787916,SRR11787927 and SRR11787938. The ChIP-seq data sets for H3K9ac were obtained from the NCBI Sequence Read Archive (SRA) database (https://www.ncbi.nlm.nih.gov/bioproject/PRJNA488988) under accession number PRJNA488988 [80], SRR8631577 and SRR8631583. The plant materials used in the study are available from the corresponding author upon request.

## Abbreviations

ATAC-seq: Assay for Transposase-Accessible Chromatin with sequencing
ChIP-seq: Chromatin ImmunoPrecipitation sequencing
EMSA: Electrophoretic Mobility Shift Assay
ACRs: Accessible Chromatin Regions
DARs: Differential Accessible Regions
PF-MARs: More Accessible Regions in the PF sample
TCP: Teosinte branched1/Cycloidea/Proliferating cell factor
ARF: Auxin Response Factor
