# Supplementary figures and images for: Integrated analysis of ATAC-seq and RNA-seq reveals the TCP-ARF molecular module related to pathogenic process of phytoplasma infection in Paulownia fortunei
Source: BMC Genomics. 2026 Mar 21;27:420. doi: 10.1186/s12864-026-12707-w (PMC13126833; doi:10.1186/s12864-026-12707-w)

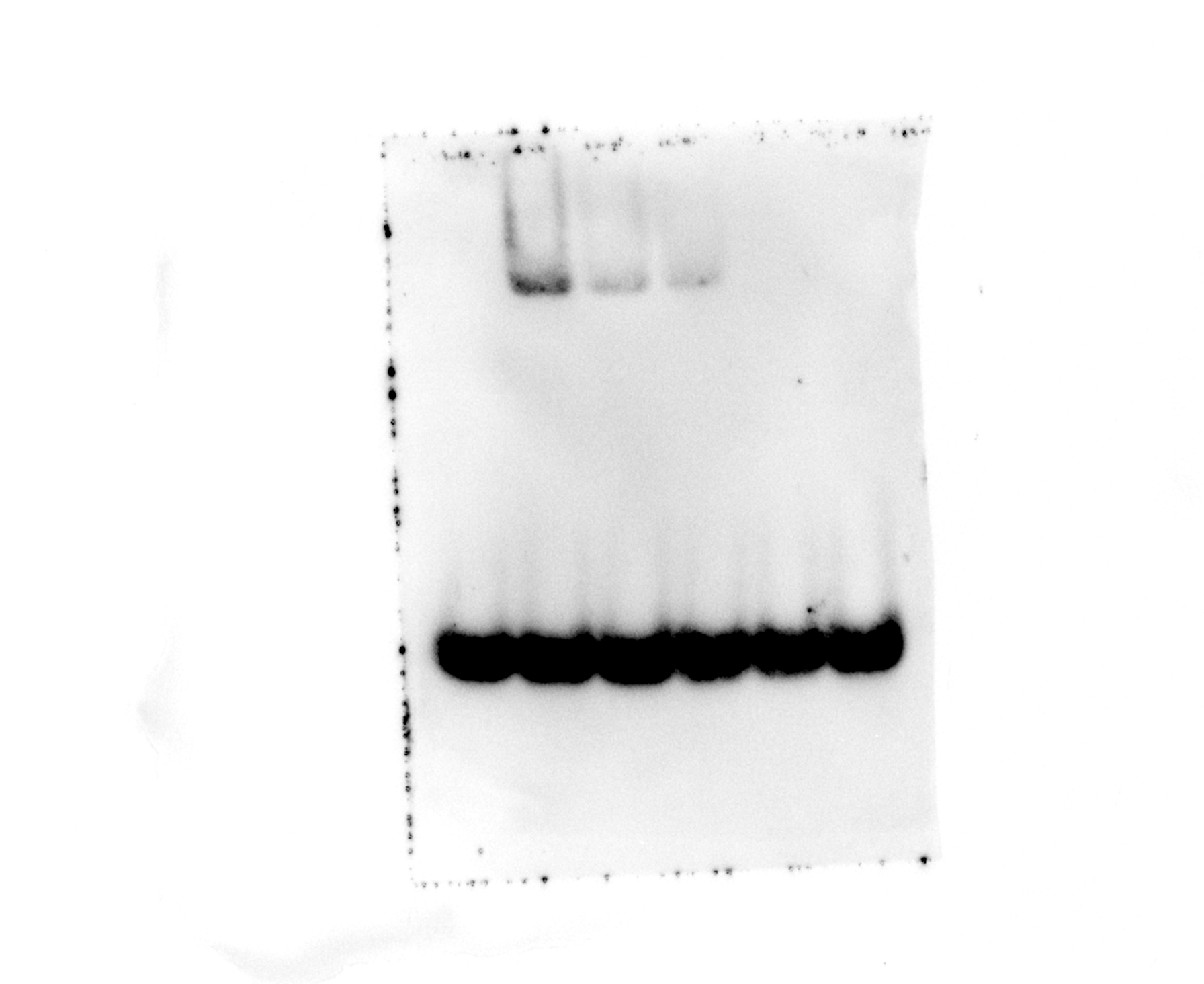

Supplement: Supplementary file 6 — Supplementary Material 6. [file 12864_2026_12707_MOESM6_ESM.tif]
